# A Data Visualization and Dissemination Resource to Support HIV Prevention and Care at the Local Level: Analysis and Uses of the AIDSVu Public Data Resource

**DOI:** 10.2196/23173

**Published:** 2020-10-23

**Authors:** Patrick Sean Sullivan, Cory Woodyatt, Chelsea Koski, Elizabeth Pembleton, Pema McGuinness, Jennifer Taussig, Alexandra Ricca, Nicole Luisi, Eve Mokotoff, Nanette Benbow, Amanda D Castel, Ann N Do, Ronald O Valdiserri, Heather Bradley, Chandni Jaggi, Daniel O'Farrell, Rebecca Filipowicz, Aaron J Siegler, James Curran, Travis H Sanchez

**Affiliations:** 1 Department of Epidemiology Rollins School of Public Health Emory University Atlanta, GA United States; 2 Oregon Health & Science University Portland, OR United States; 3 Signal Group Washington, DC United States; 4 Gilead Sciences Foster City, CA United States; 5 HIV Counts Ann Arbor, MI United States; 6 Feinberg School of Medicine Northwestern University Chicago, IL United States; 7 Department of Epidemiology Milken Institute for Public Health George Washington University Washington, DC United States; 8 Department of Epidemiology School of Public Health Georgia State University Atlanta, GA United States; 9 Maimonides Medical Center New York, NY United States; 10 Department of Behavioral Sciences and Health Education Rollins School of Public Health Emory University Atlanta, GA United States

**Keywords:** HIV, surveillance, infodemiology, data visualization, infectious disease, health policy, data dashboard, health department data, dashboard, data

## Abstract

**Background:**

AIDSVu is a public resource for visualizing HIV surveillance data and other population-based information relevant to HIV prevention, care, policy, and impact assessment.

**Objective:**

The site, AIDSVu.org, aims to make data about the US HIV epidemic widely available, easily accessible, and locally relevant to inform public health decision making.

**Methods:**

AIDSVu develops visualizations, maps, and downloadable datasets using results from HIV surveillance systems, other population-based sources of information (eg, US Census and national probability surveys), and other data developed specifically for display and dissemination through the website (eg, pre-exposure prophylaxis [PrEP] prescriptions). Other types of content are developed to translate surveillance data into summarized content for diverse audiences using infographic panels, interactive maps, local and state fact sheets, and narrative blog posts.

**Results:**

Over 10 years, AIDSVu.org has used an expanded number of data sources and has progressively provided HIV surveillance and related data at finer geographic levels, with current data resources providing HIV prevalence data down to the census tract level in many of the largest US cities. Data are available at the county level in 48 US states and at the ZIP Code level in more than 50 US cities. In 2019, over 500,000 unique users consumed AIDSVu data and resources, and HIV-related data and insights were disseminated through nearly 4,000,000 social media posts. Since AIDSVu’s inception, at least 249 peer-reviewed publications have used AIDSVu data for analyses or referenced AIDSVu resources. Data uses have included targeting of HIV testing programs, identifying areas with inequitable PrEP uptake, including maps and data in academic and community grant applications, and strategically selecting locations for new HIV treatment and care facilities to serve high-need areas.

**Conclusions:**

Surveillance data should be actively used to guide and evaluate public health programs; AIDSVu translates high-quality, population-based data about the US HIV epidemic and makes that information available in formats that are not consistently available in surveillance reports. Bringing public health surveillance data to an online resource is a democratization of data, and presenting information about the HIV epidemic in more visual formats allows diverse stakeholders to engage with, understand, and use these important public health data to inform public health decision making.

## Introduction

Public health surveillance is the basis of effective public health decision making [1], and surveillance data have been called “the conscience of an epidemic” [2]. Defined as “the ongoing, systematic collection, analysis, and interpretation of health-related data essential to planning, implementation, and evaluation of public health practice” [3], surveillance data have a critical role to play in monitoring and protecting the health of individuals and populations. More specifically, surveillance is critical to delineate patterns of disease and to evaluate population-level health data and the impact of public health interventions and policies.

Dissemination of data is a critical aspect of surveillance practice: making public health data available broadly helps to raise awareness of public health threats, promotes the efficient targeting of resources and testing programs, supports evidence-based legislation to provide for prevention and care services, and enables the monitoring of progress in disease prevention and care [4]. Historically, surveillance data have been disseminated through reports, published at least annually [5], and at varying geographic levels (ie, national, state, and local) [4]. More recently, the broad availability of the internet and increasing data transfer speeds have enabled the online dissemination of reports; in many health jurisdictions, surveillance reports are now only available online.

The US case-based surveillance system for HIV is one of the largest, most well-resourced, and comprehensive infectious disease surveillance systems in the world [6]. The US Centers for Disease Control and Prevention (CDC) administers funding for the system, which is implemented by all US states and selected US cities. The CDC is responsible for processing surveillance data and deduplicating records and for producing national surveillance reports; the CDC does not report or map HIV surveillance data at geographic levels finer than at the county level. State and local health departments are responsible for producing local surveillance reports for jurisdictions smaller than counties. Surveillance reports have traditionally largely comprised tabular presentations of data [7]. Typical surveillance reports include only summary data and are not necessarily geared to lay audiences without technical backgrounds. Data visualizations are generally lacking from surveillance reporting, although more recently, some state and local health departments have incorporated choropleth maps to illustrate variations in levels of disease by smaller geographic areas within states [8] or cities [9,10]. Despite these welcome additions, the types of data released and formats of visualizations vary across states, and the methods used to develop them may also vary among states.

We describe the development, implementation, methods, governance, and impact of AIDSVu.org, a free public data source created to visualize and disseminate HIV surveillance data. Launched in 2009, the resource is a cooperative private-public-academic partnership. We also summarize the reach and utilization of AIDSVu and its service and data resources.

## Methods

### Overview of AIDSVu and Statement of Purpose

AIDSVu was created in 2009 as an interactive online mapping platform to visualize the impact of the HIV epidemic on communities across the United States and to improve access to surveillance data at granular geographic levels. The goals of AIDSVu are to make HIV-related data widely available, easily accessible, and locally relevant to inform public health decision making. Improved accessibility and use of HIV surveillance data promotes disease awareness among the general public, media professionals, and medical practitioners and informs data-driven public health decision making. Local relevance refers to publishing data at the finest possible geographic levels, so that public health decisions can be made with awareness of heterogeneity of needs within states or cities. The platform also promotes the wide availability of multiple types of data, including surveillance data, census data on social determinants, locations of HIV-related service providers, and data on the uptake of key HIV services (eg, pre-exposure prophylaxis [PrEP] and HIV testing). Researchers have used AIDSVu’s data and tools to conduct analyses and evaluate public health interventions; in addition, advocates and community leaders have used AIDSVu’s maps and other tools to educate their audiences and support funding applications.

### Principles of Data Inclusion

With assistance from an advisory committee of key stakeholders in HIV and a technical advisory group that includes HIV surveillance technical experts, AIDSVu’s scientists have established firm principles of data inclusion to guide the scope of data to be included on the site. First, all sources must be publicly available or fully transparent to allow replication. Second, all metrics must be consistently defined and standardized to enable meaningful comparisons across geographies. Third, data presented on the site are required to be substantially population based; for example, case-based surveillance data, other data sources that represent a census of outcomes (eg, PrEP utilization data from pharmacy sources), and population-based survey data. Fourth, data must be timely; AIDSVu’s scientific team aims to provide data on the site as quickly as possible after they have been released and reviewed for data quality. Fifth, in recognition of privacy concerns and data suppression requirements set by health departments and the CDC, data are suppressed when indicated by either small numbers of cases or small populations (see Multimedia Appendix 1 for details). Within these criteria, the AIDSVu team continuously aims to release finer geographic levels of data to increase relevance to prevention and care activities in local communities.

### Overview of Data and Sources

**Figure 1:**
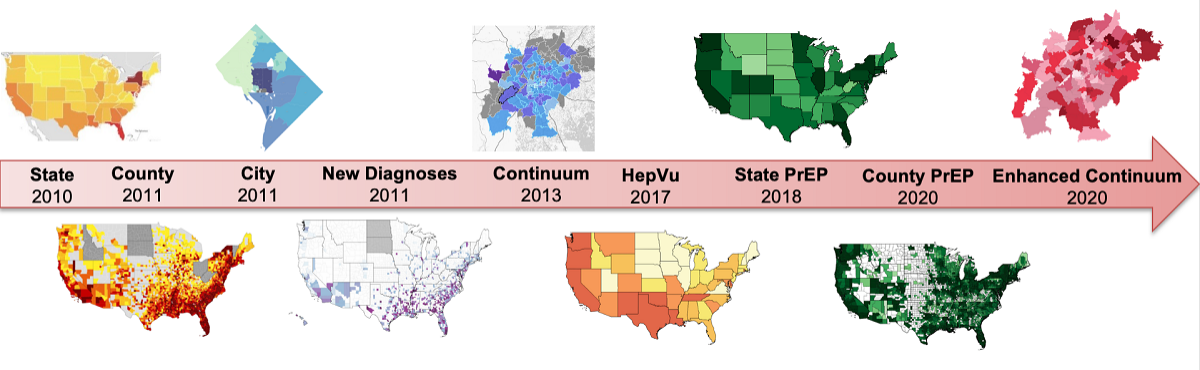
Historical progression of AIDSVu.org data presentations from 2010 to 2020. PrEP: pre-exposure prophylaxis. Hep: hepatitis C.

AIDSVu presents HIV-related metrics from a variety of sources, including the US CDC, state and local health departments, health care claims databases, and public data such as US Census data. The elements presented on AIDSVu have expanded since the platform’s inception. AIDSVu’s first HIV surveillance map released in 2010 included only state-level data, and over time AIDSVu’s team has added additional data elements at finer geographic levels, including county- and ZIP Code–level data for selected states and jurisdictions beginning in 2011 (see Figure 1).

The HIV surveillance data presented on AIDSVu are updated annually or as the data become publicly available from the data sources. HIV surveillance maps can be viewed by demographic breakdowns and can be overlaid with three types of service locations—PrEP, HIV testing, and HIV treatment—to enable examination of these resources relative to HIV prevalence and diagnosis data at different geographic levels [11].

Infographics and city and state profile pages provide supplementary visualizations and information, and complement the data presented in the maps and existing surveillance reports. Other public data, such as US Census data on social determinants of health (ie, poverty, high school education, median household income, income inequality, health insurance, etc) can be viewed alongside interactive maps to provide context. Table 1 displays the availability of data by visualization type [12,13]. Table 2 displays the availability of data by geographic level.

**Table 1:** Availability of data by visualization type from the AIDSVu Project, July 2020.

Geographic level	Profiles	Prevalence	Diagnoses	Mortality	PrEP^a^ use [12]	PrEP-to-need ratio [13]	SDH^b^	Continuum	Data sources
National	X^c^	X	X	X	X	X	X	X	CDC^d^ and Symphony Health
Regional	X	X	X	X	X	X	X		CDC
State	X	X	X	X	X	X	X	Select states	CDC
County	X	X	X	X		X	X	Select counties	CDC
ZIP Code	X	X	X				X	Select cities	City and state health departments
3-digit ZIP Code					X				Symphony Health
Census tract, neighborhood, and community area		Select cities	Select cities						City and state health departments

^a^PrEP: pre-exposure prophylaxis.

^b^SDH: social determinants of health.

^c^X: Data are presented on AIDSVu. Empty cells: data are not presented on AIDSVu.

^d^CDC: Centers for Disease Control and Prevention.

**Table 2:** Availability of data by geographic level from the AIDSVu Project, July 2020.

Available data	State	Region	County	City	ZIP Code
HIV prevalence	X^a^	X	X	Select cities	Select cities
HIV diagnoses	X	X	X	Select cities	Select cities
PrEP^b^ use	X	X	X		
PrEP-to-need ratio	X	X	X		
Mortality	X	X	X	Select cities	
Social determinants of health	X	X	X	Select cities	
Testing	X		Select counties		
Late diagnoses	X	X		Select cities	Select cities
Linkage to care	X			Select cities	Select cities
Viral suppression	X			Select cities	Select cities
Receipt of care	X			Select cities	Select cities
Federal funding	X	X			
Sexually transmitted diseases	X	X	Select counties	X	

^a^X: Data are presented on AIDSVu. Empty cells: data are not presented on AIDSVu.

^b^PrEP: pre-exposure prophylaxis.

**Figure 2:**
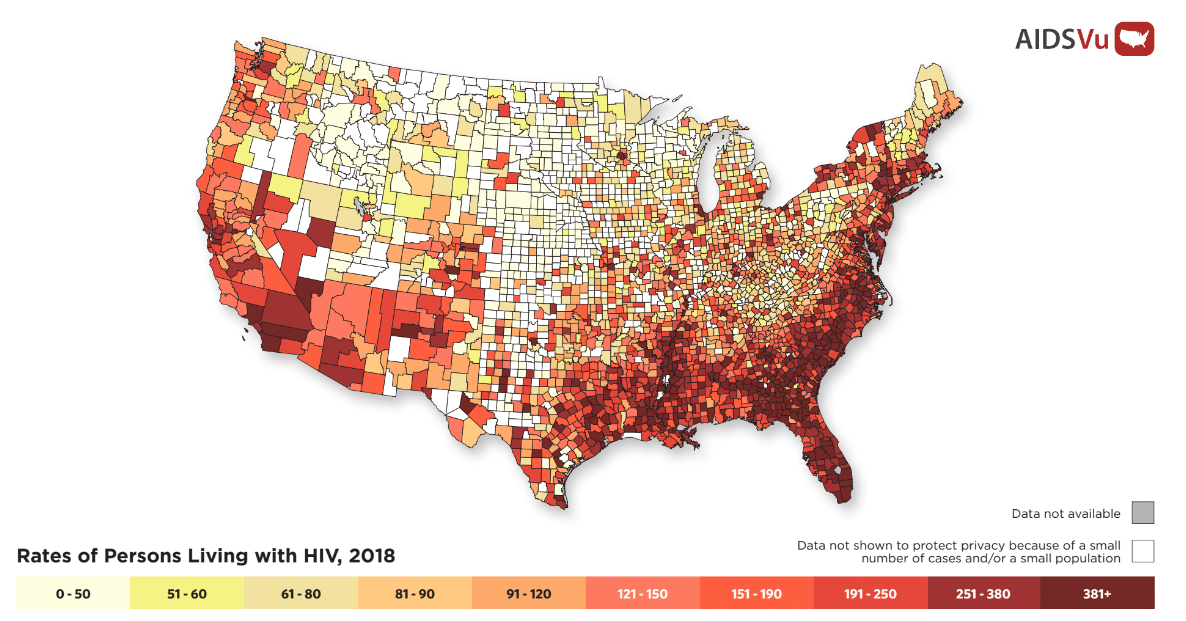
AIDSVu map of HIV prevalence at the county level, 2018.

AIDSVu currently maps HIV prevalence and new diagnoses data at state and county levels in addition to the ZIP Code level for 50 cities and community areas and wards, and maps them at the census tract level for a subset of three of those cities. At the county level, AIDSVu maps data on prevalence (see Figure 2) and new diagnoses plus PrEP use [12] and PrEP-to-need ratio [13]; at the state level, AIDSVu maps all of the prior indicators plus HIV mortality data and HIV testing data (see Table 1). For US cities, data are available at the ZIP Code level for HIV prevalence and 5-year risk of HIV diagnosis for 45 cities; data are available for the HIV continuum (eg, late diagnosis, prompt entry to care, retention in care, and viral suppression) for 35 cities.

Regarding the intended audiences, AIDSVu’s content was developed to meet the needs of several types of users: high-interest users (eg, health department staff, academic researchers, advocacy organizations, and community-based organizations), medical providers (eg, practicing clinicians and clinic staff), policy makers, the media, and the public. Different areas of the site are targeted primarily at different combinations of user audiences; for example, state profile pages might be relevant for both high-interest users and the public, data downloads are of most relevance to researchers, and infographic panels are of high relevance to all groups.

### Governance and Organizational Structure

AIDSVu is the result of a unique partnership between the academic community; governmental organizations, including federal, state, and local health agencies; and private industry. AIDSVu is led by Emory University’s Rollins School of Public Health in partnership with Gilead Sciences, Inc, and the Center for AIDS Research at Emory University. The leadership team of AIDSVu is composed of the Advisory Committee, the Technical Advisory Group (TAG), and the Prevention and Treatment Advisory Committee (PTAC), as well as a core team of staff who manage daily activities. The Advisory Committee meets annually and consists of key stakeholders who provide oversight and guidance for the project, including members from federal government agencies such as the US Department of Health and Human Services and the CDC, as well as state departments of health, academic research institutions, health-focused not-for-profit organizations, and industry. AIDSVu’s technical advisory teams (ie, TAG and PTAC) consist of HIV surveillance experts who provide input regarding data and other technical issues and HIV prevention professionals who advise AIDSVu’s team on how the tool can be utilized in HIV prevention efforts. TAG and PTAC members meet at least annually and include representatives from federal government agencies, state and city departments of health, and health-focused not-for-profit organizations. This shared responsibility of AIDSVu among public, private, and academic institutions improves ongoing sustainability, agility, and relevance to various audiences.

### Other Content Types

AIDSVu data are presented to optimize utility for the intended audiences. To do this, AIDSVu’s team provides a variety of content and visualizations, including infographics to support HIV awareness days, blog posts, downloadable map images, HIV service and clinic locators, side-by-side maps of social determinants of health data, state and local profile pages, and downloadable datasets. These have been used to support user presentations [14], research [15,16], grant applications [17], and the development of educational and advocacy materials that have been presented to stakeholders at conferences, such as the American Public Health Association, the US Conference on AIDS, the Texas HIV Prevention Conference, Fast-Track Cities, Adherence, and the International AIDS Conference.

### Awareness Day Infographics

**Figure 3:**
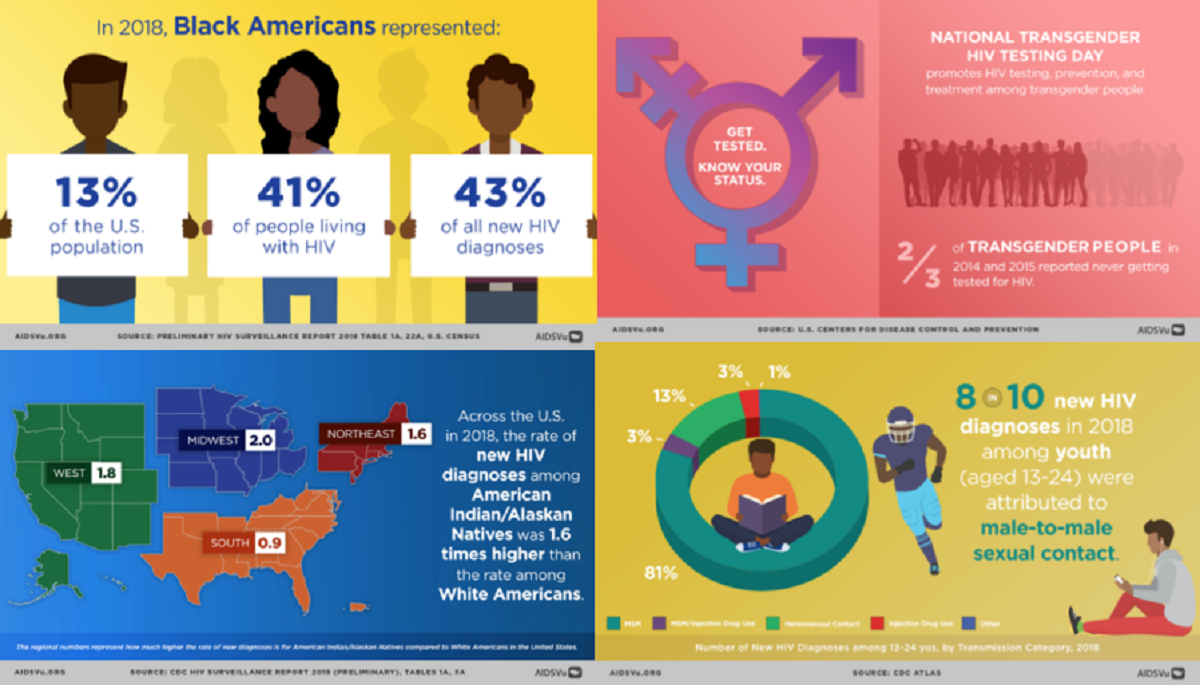
Examples of infographics for national HIV awareness days.

Annually, in observance of national HIV awareness days, AIDSVu releases a series of infographics highlighting key statistics for various populations, including gay men, women and girls, Black people, Asian and Pacific Islander populations, Native people, Latinx people, Youth, and Transgender people. These are made available to the public via Facebook, Twitter, and AIDSVu.org. The data that inform these graphics are calculated from data available on AIDSVu and from the CDC (see Figure 3).

### Blog Posts

**Figure 4:**
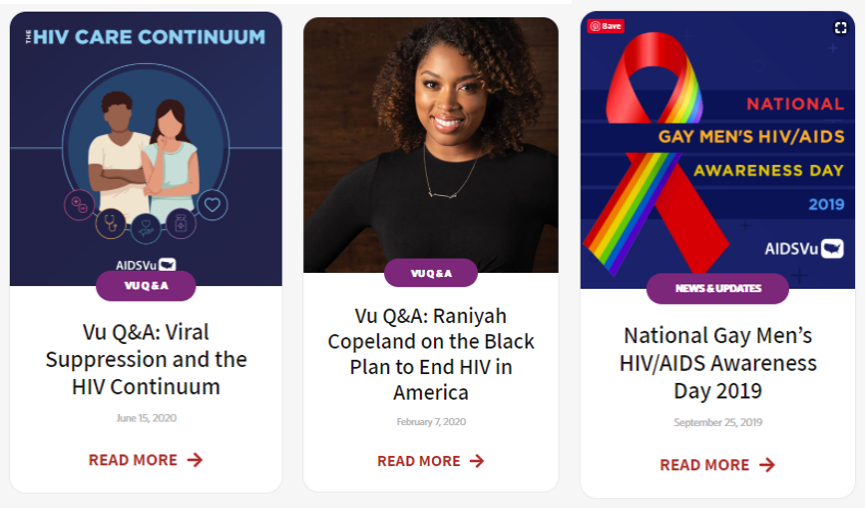
Examples of AIDSVu question-and-answer (Q&A) posts.

Throughout the course of the year, AIDSVu develops and publishes blogs posts on various topics, including HIV awareness days, the release of new data or a manuscript, a question-and-answer session on a particular issue (eg, Medicaid expansion and health inequities in the South), and years in review. Since 2010, AIDSVu has published over 160 blog posts to the website, many of which have featured some of the leading experts in HIV surveillance, research, and advocacy (see Figure 4).

### Service and Clinic Locators

**Figure 5:**
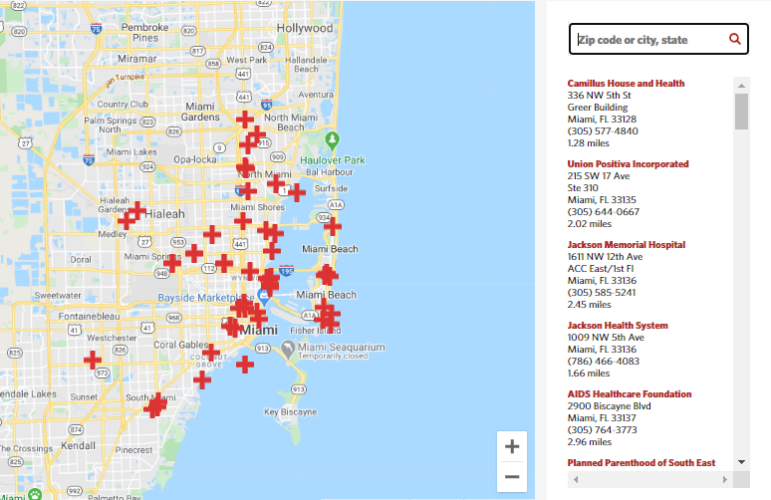
Example of HIV testing service locator for Miami Dade County.

AIDSVu provides users with service locators for HIV prevention, testing, and care. Data for testing, PrEP [18], and care services are obtained from the National Prevention Information Network through an application programming interface, so these data are as current as possible. In addition, for the nine states in the Deep South, users can locate services for stigma reduction, overdose prevention and reversal, harm reduction, and trauma-informed care (see Figure 5).

### Overlays

**Figure 6:**
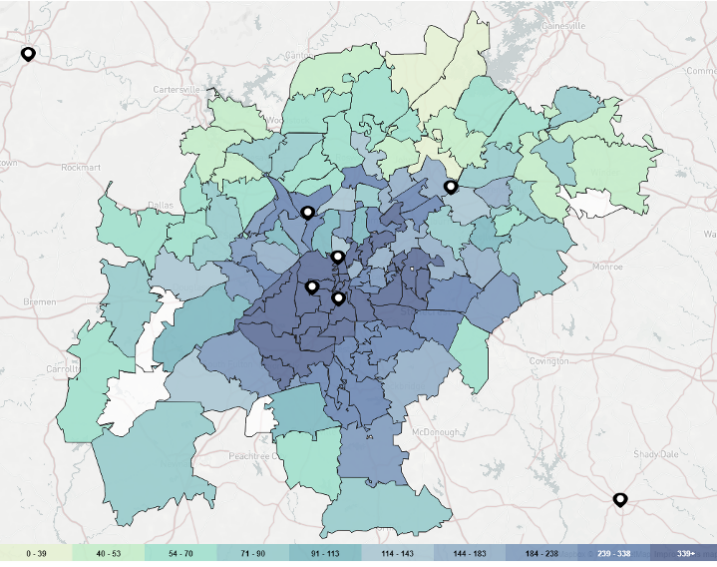
Overlay of pre-exposure prophylaxis (PrEP) services on the Atlanta ZIP Code map of the 5-year risk of new HIV diagnoses, 2014-2018.

Users are also able to overlay service provider locations directly onto AIDSVu’s maps to illustrate how services are distributed in relation to the burden of HIV. The services available for overlay include HIV testing sites, PrEP services, Ryan White HIV/AIDS Program medical care providers, the Housing Opportunities for Persons with AIDS program, National Institutes of Health (NIH)-funded HIV Prevention Trials Network sites, NIH-funded HIV Vaccine Trials Network sites, and NIH-funded HIV treatment trial sites (see Figure 6).

**Figure 7:**
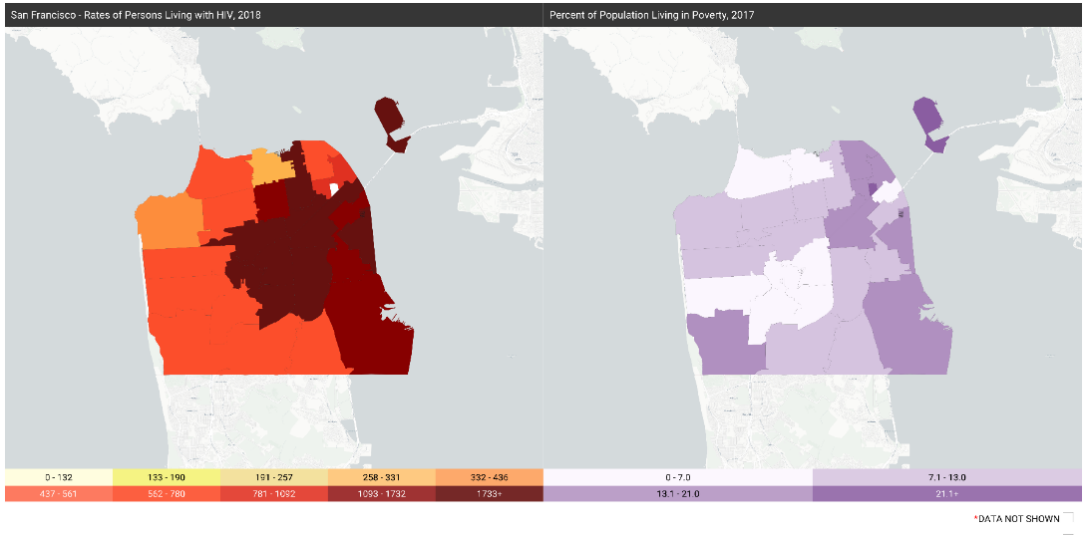
Percent of population living in poverty, San Francisco.

Social determinants of health can be displayed side by side on a secondary map to visualize the relationships between HIV and social determinants. The five social determinants included in AIDSVu are drawn from the US Census and are as follows: (1) poverty (percent of population living in poverty), (2) high school education (percent of population with a high school degree or equivalent), (3) median household income, (4) income inequality (measured by the Gini coefficient, a measure of income inequality where 0 reflects complete equality and 1 reflects complete inequality), and (5) people without health insurance (percent of population lacking health insurance) (see Figure 7).

### Profile Pages

**Figure 8:**
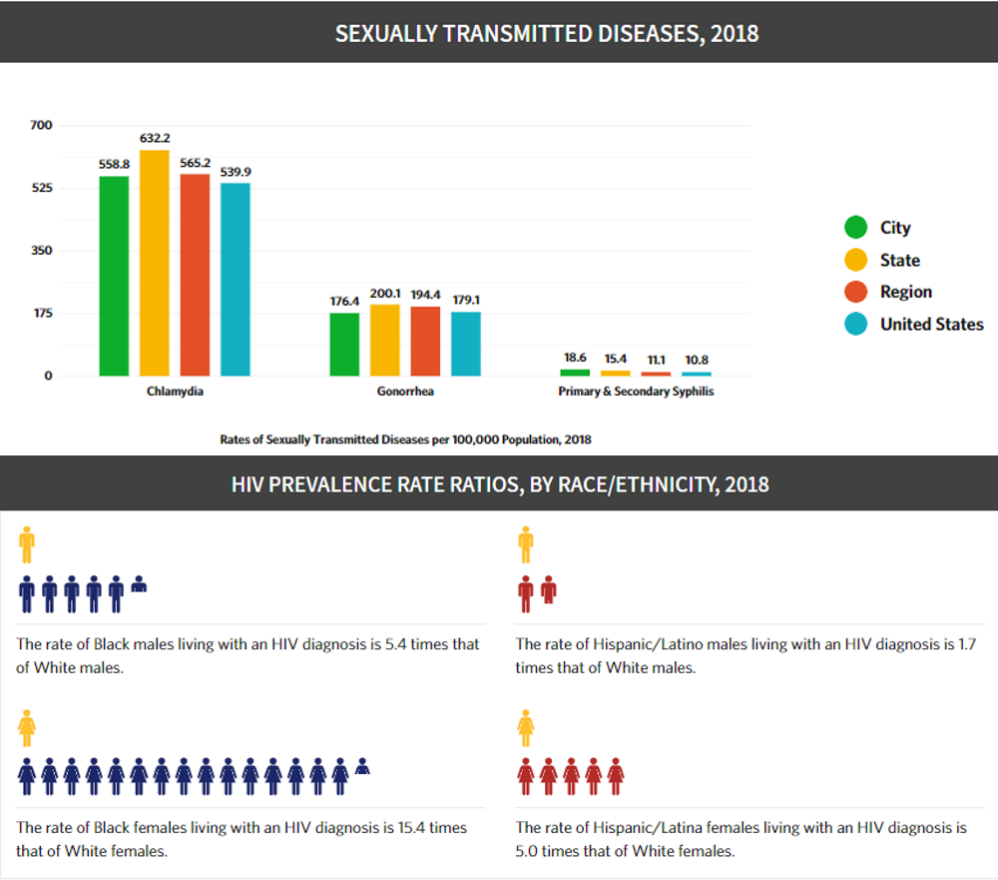
A portion of the Atlanta city profile page in AIDSVu, 2020.

The site has unique pages with profiles for over 40 US cities and 48 counties prioritized for Phase 1 of the *Ending the HIV Epidemic: A Plan for America* initiative [19]; 50 states; Washington, DC; Puerto Rico; four regions; and the nation, offering easy-to-understand, printable snapshots that summarize the impact of HIV in each of these jurisdictions. The pages enhance the maps and display data and infographics for HIV prevalence, HIV prevalence rate ratios, new HIV diagnoses, new HIV diagnoses by transmission category, HIV mortality, AIDS diagnoses, the total population, and sexually transmitted diseases data. In some cases, AIDSVu serves as a public face for data that are routinely reported by the CDC for all jurisdictions; for example, in 2018 some cities began reporting diagnosed HIV cases in male-to-female and female-to-male transgender people (see Figure 8).

### Downloadable Data and Printable Maps

Data for the overall national level, along with those at regional, state, and county levels, are available for download. Select cities also provide their data for download. The latest national, regional, state, and county data are available for HIV new diagnoses, prevalence, and mortality; social determinants of health; PrEP utilization; and PrEP-to-need ratio. PrEP data are notable because they are a resource that was developed specifically for mapping and dissemination, to meet an urgent public health need [12,20]. Users can freely download these datasets for a number of years (2008-2018) to conduct their own analyses for use in reports, publications, grant applications, etc. Users do not need to submit a data request or obtain permission to use the available data.

Similarly, users are able to download printable maps. These maps can be customized to display a toggled area, titles, and legends, and at different resolutions and transparency levels.

## Results

AIDSVu’s scientific team collects metrics to evaluate utilization over time by key audiences, including data on number of users, social media reach, and academic publications that reference AIDSVu. All data below are as of the end of 2019 unless otherwise noted.

### Website Sessions and Users

**Figure 9:**
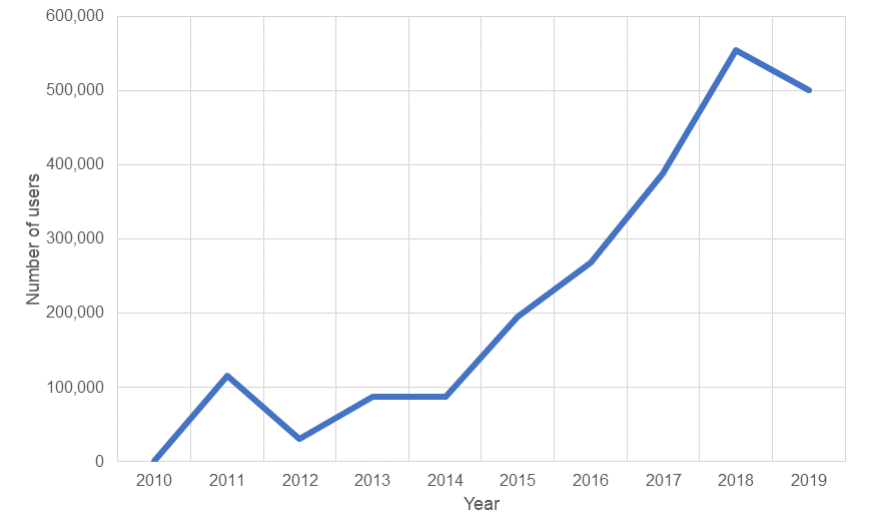
Total number of AIDSVu users by year, 2010-2019.

Since 2010 (ie, the first full year of data reporting), the number of unique website users increased from 1852 in 2010 to 501,527 in 2019 (see Figure 9). Most users (96.8%) originated in the United States, and users from five states—Georgia, Texas, Florida, California, and New York—accounted for 43.8% of all sessions in 2019; these states represented 36% of the US population in 2019.

### Social Media Reach

AIDSVu’s team also uses various social media platforms to reach key audiences. Key metrics include impressions, defined as the number of times content has been displayed on a screen, and engagements, defined as the number of actions that users take involving posts or tweets. In 2019, AIDSVu generated 3,955,972 impressions on social media—1,928,029 on Facebook and 2,027,943 on Twitter—and 113,444 engagements—48,614 on Facebook and 64,830 on Twitter. Moreover, AIDSVu’s Facebook page was followed by 10,440 users, and AIDSVu’s Twitter page was followed by 4751 users. Based on organizational types derived from IP addresses, many AIDSVu users are *high-interest* audiences (eg, health department staff, government workers, and academics). For 2019, the distribution of user types included 22,710 academic users, 5274 government users, and 6511 nonprofit users.

### Academic Publications

**Figure 10:**
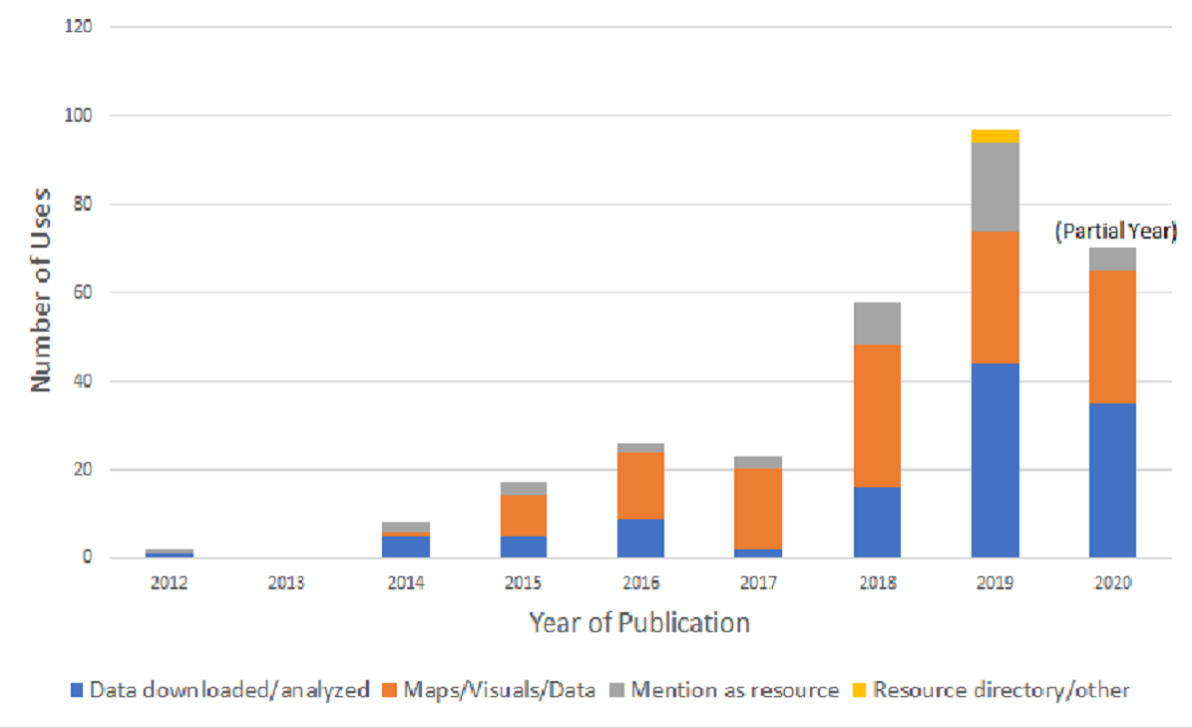
Number of academic uses of AIDSVu.org by year and use, 2012-2020.

Given that one of AIDSVu’s major objectives is to increase the availability of HIV surveillance data and related information for research purposes, the number of academic papers that cite AIDSVu is a critical metric for evaluation. As of August 22, 2020, 249 peer-reviewed journal articles, 27 academic dissertations or theses, and 6 book chapters had used AIDSVu data or cited AIDSVu’s resources. Among uses of AIDSVu in these scholarly products, common uses included use of maps or visual products (136 uses), downloading and using AIDSVu data for analyses (115 uses), citation as a public health tool (46 uses), and use of locator data for research or program purposes (3 uses). Uses of AIDSVu in academic products increased steadily from 2012 through mid-2020 (see Figure 10).

### Public Health Applications and Use Cases

Examples of how the data on AIDSVu.org are used in public health practice have been recently reviewed [11]. AIDSVu maps have been used to develop targeting for door-to-door testing campaigns, identify areas of high need for telemedicine HIV care facilities [21], and identify service gaps for HIV prevention services [11]. Funders, such as the Elton John AIDS Foundation and the Gilead COMPASS initiative, have used data on the burden of HIV diagnoses to prioritize highly impacted areas for grant funding [22]. Academic researchers have used AIDSVu data to justify the selection of study sites in grant proposals [17].

## Discussion

The US HIV surveillance system is the most comprehensive and well-funded, country-specific surveillance system for any infectious disease in the world, and the quality of the data generated by the system is unparalleled in public health surveillance activities [6]. The data have been used over the course of decades of response to the US HIV epidemic to document health disparities, to argue for resources for care, for treatment and prevention, to serve as the basis for equitable resource allocation, and to identify specific cities and facilities in need of additional resources to provide services.

Although the HIV surveillance system and quality of its data are longstanding, the development and uptake of AIDSVu is a reflection of a growing culture of access to digital data, improved computing capacity and data bandwidth to deliver dynamic content, and an emerging appetite for consuming data through visualization and interaction, in addition to, or in preference to, tabular formats. The platform builds on a substantial and well-justified public investment in surveillance data and increases the use of those data by presenting data in a way that may be more accessible to some users. This idea—the democratization of data—drives our efforts to make data on the US HIV epidemic widely available and understandable to different types of users.

One of the major enabling factors for the development and growth of AIDSVu has been the collaboration of public health colleagues at all levels of government, as detailed in the Methods section. These advisors promote appropriate uses and interpretations of data, provide advice about the best ways to display data, identify potential weaknesses in data elements or caveats to the interpretation of data, suggest emerging needs that could be met by surveillance data, and prioritize the types of information and formats that would be most useful to the public that they serve. Where possible, AIDSVu attempts to develop resources (eg, local profiles, data overlays, and map views) that reflect questions commonly asked of surveillance programs, so that routine inquiries to these programs can be referred to the website. Surveillance programs for most cities mapped by AIDSVu have agreed to release aggregated datasets for public use, fueling academic analyses and publications by facilitating ready access to data. One of the major lessons learned is that a broad collaboration of data providers and community partners has resulted in broad trust and in having data from many local health departments that are calculated using common definitions and codes and are, therefore, directly comparable to each other.

We acknowledge that surveillance data are available through multiple channels and believe that it is important that these critical data be available in many formats and through different types of partnerships. In 2012, the CDC launched the NCHHSTP (National Center for HIV/AIDS, Viral Hepatitis, STD, and TB Prevention) AtlasPlus [23], which also offers mapping capabilities at the state and county levels and custom data tabular requests. The NCHHSTP AtlasPlus also provides similar mapping and data functions for related infectious disease surveillance data, including sexually transmitted infections, tuberculosis, and viral hepatitis, which AIDSVu does not include. Conversely, AIDSVu offers some features, like ZIP Code–level mapping, HIV care continuum mapping, infographics and messaging, blog posts to facilitate data interpretation, and service provider overlays, which NCHHSTP AtlasPlus does not offer. Different platforms for presenting and facilitating the understanding of public health surveillance data are needed to serve the broad range of people who can use the data for research, program, policy, and care-planning purposes.

One of the major lessons learned in the development of AIDSVu has been the progression to displaying data at increasingly finer levels of geographic resolution. At the time of AIDSVu’s launch of county-level data in 2011, national maps of HIV case counts and prevalence were only available at the state level. The first consolidated depiction of county-level data (see Figure 2) revealed the striking heterogeneity of the impact of HIV within states, and the regional patterns of highly impacted counties in the South. The understanding of the geographic concentration within, and heterogeneity between, counties brought focus to the HIV epidemic in the US South and anticipated the national *Ending the HIV Epidemic* plan’s focus on highly impacted urban counties [19]. With the launch of ZIP Code–level data and maps on AIDSVu, geographic disparities within highly impacted cities were apparent. Some cities have agreed to map HIV prevalence data at the census tract level, providing even more precision to inform targeting of prevention and care needs.

AIDSVu has also moved to develop data sources when there was not an existing public data resource that met AIDSVu’s strict criteria for inclusion on the site. For example, AIDSVu supported the use of commercial data to develop systematic estimates of PrEP use at the state level in 2019 and accompanied the development of the data with online mapping and public-use datasets for PrEP use at the state level [12]. In 2020, AIDSVu developed and published methods to allow for county-level estimates of PrEP use, in response to the *Ending the HIV Epidemic* initiative’s programmatic focus on highly impacted counties [19,20]. This allowed for the assessment of county-level progress toward earlier goals of PrEP uptake developed under the National HIV/AIDS Strategy [24]. Further, the city-level data on AIDSVu are compiled with standardized methods and are publicly available only on AIDSVu.

The AIDSVu platform has several strengths. The strengths include the ability of the platform to provide a common platform to allow cities to display their own surveillance data. Many health departments do not have resources to build and maintain online data visualization platforms, so it makes sense to have a shared resource. Jurisdictions do not report data on ZIP Codes of reported HIV infections to the CDC, so producing city-level resources cannot be done centrally by the federal government. AIDSVu staff can develop and share code to generate local resources such as ZIP Code–level HIV continuum estimates, which is more efficient than having individual health departments develop such code. This also means that all city-level data presentations are prepared with the same methods so that data are directly comparable across cities. At the time that AIDSVu first displayed county-level data, these data were not publicly available in a single repository, but many states published their own county-level data, sometimes using different methods and assumptions. AIDSVu advanced the field in this area by making county-level estimates public that were directly comparable across state lines. AIDSVu also provides a platform to provide tools that can help interpret, visualize, and translate data to promote public health decision making (eg, map overlays to show areas in need of additional PrEP providers). Finally, AIDSVu has been a robust platform to make population-based data other than surveillance data available to researchers for academic purposes [12,25].

There are also challenges with the AIDSVu platform. The reporting of surveillance data typically lags 12-18 months between the end of a reporting period and the availability of state- and county-level data. Processing for these data includes deduplication of reports across states. In cases where health departments provide data directly to AIDSVu for city-level data displays, the cycle time is faster and data can be made available within 6-9 months, albeit without interstate deduplication and without complete ascertainment of deaths. AIDSVu has been supported for 10 years by a public-private-academic partnership, in which monetary resources are provided by Gilead Sciences and Emory University’s Center for AIDS Research; staff effort and data reporting are contributed by public health organizations. Thus, the maintenance of the website, updating of data, and development of new resources are supported with annual commitments, and are not assured in the long term.

Looking forward, AIDSVu will continue to be guided by input on the data needs and priorities of local health programs. We expect to continue moving to display more data at the most granular levels, including expansion to more US cities. Data at the city and ZIP Code level are unique data resources and will be prioritized. Similarly, we will continue to explore ways to make data on PrEP use available with more stratifications where possible, including expanding the stratification of PrEP data to include race and ethnicity. Considering other data platforms like NCHHSTP AtlasPlus, we will continue to reassess the features of AIDSVu that are value added and prioritize the development of new data resources and presentations at finer geographic levels. The AIDSVu platform, which has already been extended to map hepatitis C data [26] and SARS-CoV-2 data [27], may also be used for visualization of other diseases in the future.

Over the past decade, AIDSVu has moved to make HIV surveillance data more accessible and easy to use through innovative visualizations and tabular displays, finer levels of geography, a broad array of indicators (eg, continuum indicators and PrEP use data), and targeted dissemination for multiple stakeholders. Its impact is seen in the utilization of data resources and the growing use of AIDSVu data to support academic research and to inform policy, programmatic, and educational efforts. Surveillance data are intended to inform local responses and are most powerful when they are available for use at the city and county level.

## Appendix

Multimedia Appendix 1 Data methods for AIDVu.org as of August 2020.

## Abbreviations

CDC: Centers for Disease Control and Prevention
NCHHSTP: National Center for HIV/AIDS, Viral Hepatitis, STD, and TB Prevention
NIH: National Institutes of Health
PrEP: pre-exposure prophylaxis
PTAC: Prevention and Treatment Advisory Committee
TAG: Technical Advisory Group
