# Supplementary material for: A Data Visualization and Dissemination Resource to Support HIV Prevention and Care at the Local Level: Analysis and Uses of the AIDSVu Public Data Resource
Source: J Med Internet Res. 2020 Oct 23;22(10):e23173. doi: 10.2196/23173 (PMC7654504; doi:10.2196/23173)
Supplement: Multimedia Appendix 1 [file jmir_v22i10e23173_app1.docx]

**Online Appendix: Data Methods**

HIV Surveillance Data: State-and County-Levels

AIDSVu utilizes surveillance data from CDC to allow users to explore the HIV epidemic at state- and county-levels. Data are released to AIDSVu by CDC in accordance with HIV-surveillance-specific data re-release agreements between CDC and each state/U.S. territory health department, and in accordance with other CDC data release guidelines. Early in AIDSVu’s history, CDC did not release county-level data on behalf of state health departments, and the smallest geographic level of surveillance data available were for the state level. AIDSVu staff worked with HIV surveillance coordinators to develop a consensus around data suppression thresholds to allow re-release of county level data, and new data release policies were implemented in 2011. The HIV prevalence, new HIV diagnoses, and mortality data presented on AIDSVu are collected by state and local health departments, and de-duplicated and processed by CDC to meet data quality standards for comparability and reliability. All 50 states, the District of Columbia (DC), and U.S. territories collect comparable confidential, name-based case reports of people living with HIV infection. All diagnoses are based on an established case definition. Medical providers, laboratories, and other organizations providing HIV testing services are required, by law, to report people diagnosed with HIV to the state or local health department. Health departments report case data without names to CDC for monitoring of the national HIV epidemic.

All HIV surveillance data are displayed on AIDSVu’s maps as case counts and rates, or by proportions for transmission categories (represented as % cases). Rates are calculated per 100,000 population to permit data standardization and comparison. Denominators used to calculate rates for all state and county populations are obtained by CDC from the U.S. Census Bureau’s census for each respective year. Population denominators for all HIV surveillance data are restricted to people aged 13 and older. There are no county-level maps for Alaska and the District of Columbia because there are no counties in these states.

The data reflected on AIDSVu may slightly differ from data obtained directly from state HIV surveillance programs (e.g., on a state health department website or through a data request to a state surveillance program) because states may use analysis criteria that are different from the criteria used by AIDSVu. Further, data on AIDSVu may differ from data obtained directly from the states because AIDSVu’s data source is CDC’s National HIV Surveillance System, from which duplicate records are removed. Most recent known address was used in all analyses except for new HIV diagnoses, which are calculated based on residence at diagnosis.

HIV Surveillance Data: ZIP Code-level

In addition to the state- and county-level data described above, AIDSVu also displays ZIP Code-level data for over 40 jurisdictions (i.e., cities or Metropolitan Statistical Areas or select counties or ZIP Codes), with a few cities displaying census tract- and/or community area/ward-level data as well. To permit the display of HIV prevalence and new HIV diagnoses data by ZIP Code, AIDSVu obtains an annual data release agreement with state or local health departments overseeing HIV surveillance in participating cities. Data are obtained directly from the state or local public health departments rather than from CDC because CDC does not collect street addresses for reported cases due to privacy concerns. ZIP Code-level data are not directly comparable to the state- and county-level HIV data displayed on AIDSVu because the data are not obtained directly from CDC. Each city, county, or state public health department defines the geographic area (e.g., ZIP Codes) in their jurisdiction for which they desire to display data on AIDSVu. HIV prevalence is reported as one-year prevalence rates and case counts. HIV diagnoses case counts are reported as 5-year counts and rates of new diagnoses (e.g., 5-year risk of HIV diagnosis) to allow display of data at the Zip Code-level without excessive suppression of data due to small numbers of diagnoses.

Data Suppression and Rate Stability

For HIV surveillance data, AIDSVu developed and applied a set of data suppression rules. To protect the privacy of persons living with HIV infection, AIDSVu does not display rates and case counts for states and/or counties when one or more of the following suppression criteria are met:

1) Numerator (number of persons living with HIV infection) is greater than zero and less than five at the county-level and/or the denominator (number of people in the county in that population group) is less than 100. If the overall county case count is greater than zero and less than five and/or the overall county population is less than 100, all data for the county are suppressed.

2) For breakdowns by sex at the county-level, if either the male or female rate and/or case count is suppressed because of the numerator/denominator thresholds mentioned in #1, both sex groups are suppressed to prevent indirect identification.

3) For breakdowns by age at the county level, if the rate and/or case count for only one age group is suppressed in a county because of the numerator/denominator thresholds mentioned in #1, then the 13-24 age group is also suppressed to prevent indirect identification. If the 13-24 age group is the only age group suppressed for a county, then the 55+ age group is also suppressed.

4) If data for only one county in a state is suppressed, then one additional county is also suppressed, with the additional county selected based on having the smallest total population of the remaining counties. In the event there are multiple counties in a state with the exact same total population, the county with the lowest number of HIV cases is also suppressed. In the event there are multiple counties in a state with the same total population and the same total number of HIV cases, then both or all of these counties are suppressed.

States and counties are noted by the color white on the AIDSVu map when one or more of these conditions are met. As is standard in the display of health statistics, rates generated from a numerator less than 12 are considered unstable and should be interpreted with caution. Because rates at the county level are not displayed when the numerator is greater than zero and less than five, the “unreliable rate” indicator will only display when the numerator is zero, five or greater and less than 12. Data are suppressed in accordance with the state- and county-level data suppression requirements approved by each state under a data re-release agreement with CDC. States may choose to suppress cases/rates for counties with a population below a specified threshold at the overall level, or for specific stratification levels. States may also choose to suppress data at the state-level overall, or for specific stratification levels. The data for those states and counties are not released to AIDSVu and appear gray on the map. For year-by-year new HIV diagnoses data, if the overall county case count breakdown is greater than zero and less than five, all data for the county are suppressed.

To protect the confidentiality of persons living with diagnosed HIV in displayed cities at ZIP Code-level, AIDSVu does not display rates and case counts when the numerator (number of persons living with HIV) is less than five and/or the denominator (number of people in the ZIP Code in that population group) is less than 500. ZIP Codes are noted by the color white when one or both of these conditions are met (see footnote below map scale). Philadelphia and Seattle are the only exceptions where the data are suppressed when the numerator is less than six. As is standard in the display of health statistics, rates generated from a numerator less than 12 are considered unstable and should be interpreted with caution. Because rates are not displayed when the numerator is less than five, the “unstable rate” indicator will only display when the numerator is five or greater and less than 12.

To protect the privacy of persons using PrEP, AIDSVu will not display PrEP data for states or at the ZIP3 unit-level when one or more of the following suppression criteria are met:

1) The number of persons using PrEP is less than three at the ZIP3 unit-level and/or the denominator (number of people in the ZIP3 unit in that population group) is less than 100. If the overall ZIP3 unit has less than three PrEP users and/or the overall ZIP3 unit population is less than 100, all data for the ZIP3 unit are suppressed.

2) For breakdowns by sex at the ZIP3 unit-level, if either the male or female PrEP data are suppressed because of the numerator/denominator thresholds mentioned in #1, both sex groups are suppressed to prevent indirect identification.

3) For breakdowns by age at the ZIP3 unit-level, if the PrEP data for only one age group are suppressed in a ZIP3 unit because of the numerator/denominator thresholds mentioned in #1, then the ≤24 age group is also suppressed to prevent indirect identification. If the ≤24 age group is the only age group suppressed for a ZIP3 unit, then the 55+ age group is also suppressed.

4) If data for only one ZIP3 unit in a state is suppressed, then one additional ZIP3 unit is also suppressed, with the additional ZIP3 unit selected based on having the smallest total population of the remaining ZIP3 unit.

States and ZIP3s are noted by the color white on the AIDSVu maps when one or more of these conditions are met. Rates generated from a numerator less than 12 are considered unstable and should be interpreted with caution. Because rates at the ZIP3-level are not displayed when the numerator is less than three, the “unreliable rate” indicator will only display when the numerator is three or greater and less than 12.

Due to suppression criteria for PrEP users and new diagnoses cases, AIDSVu will not display PNR data for counties when one or more of the following suppression criteria are met:

1. If PrEP users or newly diagnosed cases are suppressed for a county based on the criteria mentioned above, then the PNR for that county is also suppressed.
2. If the number of newly diagnosed cases for a county is 0, then the PNR for that county is suppressed.

Social Determinants of Health

The five social determinants mapped for both state- and county-level data are: poverty (percent of population living in poverty), high school education (percent of population with a high school degree or equivalent), median household income, income inequality (measured by the Gini Coefficient, a measure of income inequality where 0 reflects complete equality and 1 reflects complete inequality), and people without health insurance (percent of population lacking health insurance). All SDOH data are obtained from the US Census Bureau.

HIV PrEP Data

In 2018, AIDSVu released the first publicly available HIV PrEP (TDF/FTC) annual utilization data (“PrEP use”) by state and ZIP3. The data were obtained from Symphony Health through a partnership with Gilead Sciences, Inc., and compiled by AIDSVu’s scientific team at Emory University. Symphony Health provided Gilead with national, electronic, patient-level prescription data from an overall sample that represents more than 54,000 pharmacies, 1,500 hospitals, 800 outpatient facilities, and 80,000 physician practices across the U.S. All patient-level prescription data were de-identified and linked to confirmatory data from a de-identified medical insurance claims database. The methods calculation of metrics for PrEP utilization have been described.

PrEP data are displayed on AIDSVu as the number of PrEP users and rates of PrEP use. The data represent the number of people who had at least one day of prescribed TDF/FTC for PrEP in a calendar year from 2012 to 2018. Rates of PrEP use are calculated per 100,000 population to permit data standardization and comparison. The American Community Survey (ACS) one-year population estimates are used for the denominator for yearly state-level data (e.g., 2017 population estimates are the most current year available and were therefore used for the denominator for 2018 PrEP rates). ACS combined five-year population estimates (2012-2016) are used for the denominator for yearly PrEP use at the ZIP3-level. Denominator totals are for ages 13+, which were obtained from the ACS data by taking the 10-14 age grouping and multiplying by 2/5ths to estimate 13-14-year-olds and then adding with all the other age groups. The denominator totals for the ≤24 age grouping was developed by combining 13-14 (again obtained by multiplying the 10-14 age grouping by 2/5ths), 15-19 and 18-24, to create one age category of 24 or less.

The PrEP-to-Need Ratio (PNR) data compare the ratio of the number of PrEP users from 2012-2018 to the number of people newly diagnosed with HIV in each corresponding year. The numerator is the number of PrEP users, by year, and the denominator is new HIV diagnoses cases, by year. Because 2017 new diagnoses is the latest year available on AIDSVu, it is used to calculate 2018 PNR.

Data Stratification

AIDSVu presents various data stratifications to provide more detail to the user. AIDSVu allows viewers to look at HIV surveillance data at the overall geographic level, and by race/ethnicity, sex, and age groups. The Black, White, and Asian race groups are exclusive of Hispanic/LatinX people, and the Hispanic/Latinx ethnicity is inclusive of all races. Additionally, AIDSVu displays transmission categories with two-way stratification possibilities at the state level for both prevalence and new HIV diagnoses and displays one-way stratification possibilities at the state level for mortality. State-level two-way stratifications include age*sex, age*race, age*transmission category, sex*race, sex*transmission category, and race*transmission category for prevalence and new diagnoses. Note that transmission category includes MSM and MSM/IDU, which already encompases male+transmission category. State-level mortality and county-level prevalence also include sex*transmission category. Data have been statistically adjusted to account for missing transmission category (see CDC methodology for using multiple imputation to assign a transmission category). The county-level HIV prevalence data include one-way transmission categories in addition to the other demographic data. All race groups are non-Hispanic, and the Hispanic/Latinx ethnicity is inclusive of all races. Cases are assigned to age groups based on “age at the end of 2017” (the year for defining birth is based on the most recent available data on HIV diagnoses).

The PrEP and PNR data can be stratified by age and sex but cannot currently be stratified by race/ethnicity. Age is defined by “year at birth” and displayed as 24 and under (13-24), 25 to 34, 35 to 44, 45 to 54, 55+. Age is defined by year of birth and displayed as 24 and under (13-24), 25 to 34, 35 to 44, 45 to 54, 55+.

AIDSVu allows viewers to look at ZIP Code-level HIV rates and case counts of prevalence data and five-year combined cases of new HIV diagnoses data at the overall geographic level, and by race/ethnicity, sex, and age groups.

Data Caveats

*HIV Surveillance Data:* There are various caveats that should be noted for the HIV surveillance data on AIDSVu. First, HIV surveillance data are displayed on AIDSVu only for Black, White, and Hispanic/Latinx persons at the county level because data for Asian, Native Hawaiian/Other Pacific Islander, Multiple Races, and American Indian/Alaska Native persons do not meet CDC’s criteria for statistical reliability, data quality, or confidentiality due to small population denominators or small HIV case counts. Race/ethnicity data for Puerto Rico is displayed only by case counts and not by rate. Second, transmission category stratification displays cases and percent cases (proportions) for all transmission categories. In addition, rates of HIV infection among men who have sex with men (MSM) can also be viewed at state-level for HIV prevalence, new diagnoses, and mortality and at county-level for prevalence. These rates were developed utilizing the first population estimates of MSM in every state and county in the U.S . [^31^](https://paperpile.com/c/uIbDaz/IpGW) The estimates were used as the denominator and the rates are per 100 MSM. Due to a lack of population estimates for injection drug use (IDU), IDU/MSM, and heterosexual male and female populations, rates for the transmission categories aside from MSM are unable to be calculated or displayed. Finally, new HIV diagnoses data are displayed only for overall rates and cases and not for any race/ethnicity, sex, or age groups at the county level.

Data caveats for ZIP Code data are unique to each city and are outlined on the Data Methods section on the website.

There is currently no single entity or data source that collects data on all users of PrEP across the U.S. Symphony Health collects data from more than 54,000 pharmacies, 1,500 hospitals, 800 outpatient facilities, and 80,000 physician practices across the U.S. This is an open sample of commercially available data, which includes prescription, medical, and hospital claims data for all payment types, including commercial plans, Medicare Part D, cash, assistance programs, and Medicaid. The dataset also includes data from some clinics in academic settings. However, the dataset does not contain all sources of TDF/FTC prescriptions in the U.S. For example, closed healthcare systems do not share their data with Symphony Health. Additionally, other entities may choose not to share their data with Symphony Health for their own reasons. AIDSVu’s PrEP dataset also excludes TDF/FTC prescriptions that do not have sufficient medical procedure or diagnosis codes to confirm that the prescription was for PrEP and not for any other use, such as HIV treatment, chronic Hepatitis B treatment, or post-exposure prophylaxis. To account for underestimations of PrEP use due to misclassified prescriptions, the county-level PrEP users data were upweighted by using state-specific percentages of unclassified prescriptions (prescriptions with an unknown indication) to create the AIDSVu weighted PrEP data. The unrounded and unsuppressed number of weighted PrEP users per county were then summed by state to obtain state-, regional-, and national-level estimates. The actual number of people prescribed PrEP is higher. All new state-, regional-, and national-level data are now the sums of the county-level data. State-level data previously provided on AIDSVu may slightly vary from the new state data obtained by summing up county PrEP to the state-level. The minor differences are attributed to missingness of geolocation variables and difference in data cleaning algorithms when extracting data at the state versus at the ZIP3 level (from which the county-level data is derived).

Data are derived from prescriptions to unique people; however, those who fill a prescription may not use it. The overall total population may be fewer than the sum of age group total population for a given year because people may be counted twice if they switch age groups within a certain year (i.e. if a person turns 35 in 2018 then the person is counted in both the 25-34 and 35-44 age groups in 2018). Additionally, the number of PrEP users by sex and age may not perfectly sum up to overall estimates due to differences in sex- and age-specific weights applied to the raw data. At the time the 2018 PNR data were calculated, 2018 PrEP data and 2017 HIV New Diagnoses were the most current datasets available. Therefore, 2017 New Diagnoses data were used as the denominator when calculating 2018 PNR.

Ranges/Legend Values

Range intervals were initially developed using deciles in SAS analytic software (SAS Institute, Cary, NC). Set cut points were derived using rounded deciles from the previous year, when previous year data were available. When previous year deciles were not available, rounded current year deciles were used for data new to this release. In order to illustrate the variation in the data, ranges were developed specific to each geographic level (i.e., states or counties), for each one-way grouping (i.e., overall, separately by race, by sex, by age group and by transmission category), and for each two-way group combination (i.e., by sex and age group, by sex and race, by sex and transmission category, etc.), when data were available.

Thus, for state prevalence and new HIV diagnoses maps, a total of 23 sets of map scales exist for each (rate/count by overall/race/sex/age/two-way, proportion/case by transmission category and age/transmission category and sex/transmission category and race, and transmission category total case counts and proportions for heterosexual, IDU, and other). For state mortality, there are 18 sets of scales (rate/count by overall/race/sex/age and proportion/case by male/female transmission category, and transmission category total case counts and proportions for heterosexual, IDU, and other). A total of 17 sets of map scales exist for county-level prevalence data (rate/count by overall/race/sex/age and proportion/case by male/female transmission category and overall transmission category). For county-level new HIV diagnoses maps, a total of two map scales exist (rate/count by overall). A total of six map scales each exist for state- and county-level PrEP data (users/rates by overall/sex/age). For state- and county-level PNR maps, a total of three maps exist for each (ratio by overall/sex/age).

For each scale calculation, the original deciles used to create the rounded set cut points are determined by combining the individual rates or counts for all areas with data included on the maps for that geographic level and demographic grouping.

Caution should be exercised when viewing and interpreting different maps because the scales change across the different demographic breakdowns and geographic levels.

Corrections Note

The HIV prevalence and new diagnoses data displayed on AIDSVu include state and federal correctional populations. Their inclusion may artificially inflate the HIV prevalence rate and case count of counties that house institutions. A correctional warning is displayed in the map hover-over balloon of certain counties when artificial inflation may be present based on predefined criteria. To determine in which counties the correctional warning should appear, the following process and predefined thresholds were used to categorize counties.

1. All U.S. counties were categorized by level of urbanization using the National Center for Health Statistics (NCHS) Urban-Rural Classification Scheme.
2. Federal and state correctional populations were obtained for each county from the 2010 U.S. Census.
3. Within these strata of urbanization, counties are assigned the corrections warning if they met the following criteria:

a. Above average HIV prevalence rate (as compared to counties with no correctional population), and

b. Above average correctional population or above average percent of the population housed in state/federal correctional institutions.
